# Efficacy and safety of anlotinib-based treatment in metastatic breast cancer patients

**DOI:** 10.3389/fonc.2022.1042451

**Published:** 2022-12-09

**Authors:** Yu Qian, Kexin Lou, Hao Zhou, Lili Zhang, Yuan Yuan

**Affiliations:** ^1^ Department of Oncology, The Affiliated Cancer Hospital of Nanjing Medical University, Nanjing, China; ^2^ Department of Pathology, The Affiliated Cancer Hospital of Nanjing Medical University, Nanjing, China; ^3^ Department of Chemotherapy, Jiangsu Cancer Hospital, Jiangsu Institute of Cancer Research, The Affiliated Cancer Hospital of Nanjing Medical University, Nanjing, China

**Keywords:** metastatic breast cancer, anlotinib, antiangiogenesis, efficacy, side effect

## Abstract

**Objective:**

To evaluate the efficacy and safety of anlotinib-based treatment in metastatic breast cancer (MBC) patients with failure of standard treatment.

**Methods:**

We collected the medical data of 56 female patients with the diagnosis of MBC and had failed the standard treatment before. These patients received at least two cycles of anlotinib-based treatment as the second-line or beyond treatment between October 2019 and April 2022 in Jiangsu Cancer Hospital. The primary endpoint of our study was progression-free survival (PFS), and it was estimated with Kaplan-Meier. The second end points were disease control rate (DCR), objective response rate (ORR), and side effects.

**Results:**

The median PFS time of a total of 56 patients was 5.7 months (95% CI, 3.17-8.23 months). The ORR and DCR was 28.6% and 71.4%, respectively. In second-line, third-line, and beyond treatment, the median PFS was 11.7 months, 8.7 months, and 4.7 months, respectively. In different subtype of breast cancer, the median PFS was 5.6 months, 5.7months, and 6.4 months in human epidermal growth factor receptor 2 positive (HER2+), hormone receptor positive and HER2 negative (HR+/HER2-), and triple negative breast cancer (TNBC) patients, respectively. Most adverse effects were clinically manageable, and the most common events were platelet count decrease (35.7%), hand-foot syndrome (19.6%), diarrhea (19.6%), and fatigue (17.9%). The most common grade 3 and 4 adverse events were platelet count decrease (10.7%), diarrhea (7.1%), and oral mucositis (5.4%).

**Conclusion:**

Anlotinib-based treatment showed good efficacy and manageable toxicity in multi-line treatment of MBC patients who failed the standard treatment.

## Introduction

Breast cancer is now the most common malignant tumor and the leading cause of cancer-related mortality in women. The 5-year relative survival rate for breast cancer is 90%, however, 30-40% of early-stage breast cancer patients still experience recurrence and metastasis, becoming incurable metastatic breast cancer (MBC) (1, 2). With the emerging targeted therapies such as CDK4/6 inhibitors, mTOR inhibitors, and anti-HER2 targeted therapy, the prognosis of breast cancer patients has been improved. Nevertheless, for patients with MBC and have received first- or second-line standard treatment, there is currently no standard treatment guideline for them. Treatment decisions in this setting have a great impact on the prognosis of the patients.

Breast cancer is an angiogenesis-dependent tumor (3), the rapid growth of the tumor requires sufficient blood supply to provide nutrients. Folkman proposed that angiogenesis is not only a prerequisite for tumor growth but also an essential factor in promoting tumor metastasis, we can inhibit tumor growth by inhibiting angiogenesis (4, 5). Vascular endothelial growth factor (VEGF) is essential for developing the vascular system at the early stage of the tumor and plays a crucial role in tumor proliferation and metastasis. The expression of VEGF in breast cancer tissue is 7 times higher than in normal tissue (6, 7). VEGF secreted by cancer cells acts on the vascular endothelial growth factor receptors (VEGFR) of vascular endothelial cells in the adjacent stroma, promotes the division and proliferation of vascular endothelial cells, induces tumor angiogenesis, and increases vascular permeability. It can also activate VEGFR-mediated downstream signal transduction. In addition, VEGF produced by cancer cells acts on the VEGF/VEGFR autocrine loop to promote cancer cell proliferation and facilitate evasion of apoptosis. The inhibition of tumor angiogenesis is an important mechanism and target of breast cancer anti-tumor therapy (7). The main categories of VEGF targeted therapy are currently monoclonal antibodies and small-molecule antiangiogenic tyrosine kinase inhibitors (TKIs). Monoclonal antibodies represented by bevacizumab showed conflicting results in clinical trials while the efficacy of anti-angiogenic TKIs also remains controversial. The role of anti-angiogenic therapy in breast cancer is still unclear.

Anlotinib is a new type of small-molecule antiangiogenic tyrosine kinase inhibitor that targets VEGFR, fibroblast growth factor receptor (FGFR), platelet-derived growth factor receptors (PDGFR), and c-kit (8). Anlotinib inhibits cell migration and capillary-like tube formation, and angiogenesis induced by VEGF.Anlotinib also decreased the expression of proangiogenic factors, enhanced the expression of immune cell adhesion molecules and chemokines and their receptors. It suppressed tumor angiogenesis and normalized the remaining blood vessels (8, 9). It has shown significant efficacy and been approved for indication in some malignant carcinomas such as advanced non-small cell lung cancer (NSCLC) and advanced soft tissue sarcoma in China (10–12). Professor Yuan had presented the good results of the treatment of anlotinib in advanced HER2 negative breast cancer in the SABCS meeting in 2019 (13). Based on these previous studies, we explored the use of anlotinib-based treatment in breast cancer patients who had failed standard therapy. In this study, we retrospectively analyzed the efficacy and safety of anlotinib-based treatment in these patients.

## Methods

### Data source and study population

This study was a retrospective single-center analysis conducted in the Jiangsu Cancer Hospital. MBC patients with the failure of standard treatment and received anlotinib-based treatment at our hospital as the second-line or beyond treatment between October 2019 and April 2022 were enrolled. We collected the medical data of 56 female patients. These patients were aged 34-76 years old with Eastern Cooperative Oncology Group (ECOG) performance status of 0-2.

### Treatment methods

Patients used anlotinib in combination with chemotherapy or immunotherapy. Chemotherapy drugs mainly include capecitabine, nab-paclitaxel and vinorelbine. Capecitabine was given at 1000mg/m2 twice daily for 14 days on followed by 7 days off, nab-paclitaxel was given at 125 mg/m2 on days 1 and 8, and vinorelbine was given at 25 mg/m2 on days 1 and 8. We mainly use tislelizumab, an anti-human programmed cell death 1 (PD-1) monoclonal IgG4 antibody, at the dosage of 200 mg every 3 weeks for immunotherapy. HER2-targeting therapies were also used in HER2-positive breast cancer patients. All patients received anlotinib at a dosage of 12 mg, 10 mg, or 8 mg at the beginning. Anlotinib is an oral small molecule inhibitor of multiple receptor tyrosine kinases that is being co-developed by Jiangsu Chia-Tai Tianqing Pharmaceutical and Advenchen Laboratories for the treatment of advanced cancer (14). Anlotinib is available in three dose levels of 12 mg, 10 mg, and 8 mg. The recommended dosage of anlotinib is 12 mg once daily taken orally continuously for 2 weeks on and one week off, 21 days as one cycle. For patients with good general conditions and few adverse reactions to previous chemotherapy, physicians would use a standard dose (12 mg) of anlotinib. For patients with poor general conditions and significant adverse reactions to prior chemotherapy, considering that these patients may not be able to tolerate the 12mg dose, the physician would choose a 10mg or 8mg dose of the drug according to the specific situation the patient. Doses modifications were allowed when patients experienced drug-related toxicity (14, 15).Treatment was continued until disease progression or unacceptable toxicity.

### Evaluation of efficacy and safety

Treatment response was assessed every two cycles according to the Response Evaluation Criteria in Solid Tumors (RECIST 1.1). The primary endpoint of our study was progression-free survival (PFS), and it was defined as the time from the onset of anlotinib to disease progression or death. Other evaluations of the therapeutic effects included the objective response rate (ORR) and disease control rate (DCR). Complete response (CR) was defined as complete resolution of all target foci; partial response (PR) refers to conditions with a ≥30% length reduction for the baseline foci; progression of disease (PD) refers to conditions with a total length increase of ≥20% for the baseline foci or the appearance of one or more new foci(s); stable disease (SD) refers to conditions between PD and PR. ORR was defined as the proportion of patients who had a PR or CR to therapy. DCR was defined as the proportion of patients who achieved CR, PR, and SD in response to therapy. Adverse events were evaluated according to the Common Terminology Criteria for Adverse Events version 5.0 (CTCAE 5.0).

### Ethics statement

The study was conducted in accordance with the Declaration of Helsinki (as revised in 2013). The study was approved by ethics board of Jiangsu Cancer Hospital (No. 2020-042), and individual consent for this retrospective analysis was waived.

### Statistical analysis

PFS was estimated using the Kaplan-Meier method, and comparisons were calculated using the log-rank test. Data processing and analysis were performed using SPSS version 26.0 (IBM, New York, USA). The significance was accepted when P<0.05.

## Results

### Patient characteristics

The median age of a total of 56 patients was 53 years. Among them, 14 patients (25.0%) had histologically confirmed HER2+, 15 patients (26.8%) were HR+/HER2-, and 27 patients (48.2%) were diagnosed with TNBC. Anlotinib-based treatment was used in second-line, third-line, and beyond treatment in 11 (19.6%), 15 (26.8%), and 30 (53.6%) patients, respectively. All patients received taxane in the (neo)adjuvant or metastatic setting. The median number of treatment lines was 4. Anlotinib was used in combination with chemotherapy or immunotherapy. A summary of patients’ characteristics was shown in **Table 1**.

**Table 1:** Baseline characteristics of 56 patients.

Characteristics	Patients(n=56)
**Age, years, median (range)**	53 (34-76)
**Molecular subtyping**	
HER2+	14 (25.0%)
HER2-	42 (75.0%)
HR+/HER2-	15 (26.8%)
TNBC	27 (48.2%)
**Treatment Line, median (range)**	4 (2-8)
2	11 (19.6%)
3	15 (26.8%)
≥4	30 (53.6%)
**Disease type**	
Visceral	43 (76.8%)
Non-visceral	13 (23.2%)
**Metastatic sites**	
Liver	22 (39.3%)
Lung	25 (44.6%)
Brain	15 (26.8%)
Bone	25 (44.6%)
Pleura	12 (21.4%)
**Prior therapies**	
Prior anthracyclines	49 (87.5%)
Prior taxane	56 (100%)
**Treatment regimens**	
Combined with chemotherapy	51 (91.1%)
Capecitabine	16 (28.6%)
Taxane	15 (26.8%)
Vinorelbine	9 (16.1%)
Combined with immunotherapy	5 (8.9%)
**Starting dose**	
12 mg	34 (60.7%)
10 mg	18 (32.1%)
8 mg	4 (7.1%)

### Efficacy

**Figure 1:**
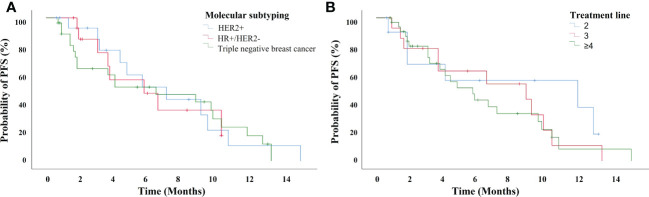
The progression-free survival for 56 breast cancer patients treated with anlotinib-based treatment **(A)** with different subtyping, and **(B)** in different treatment lines.

**Figure 2:**
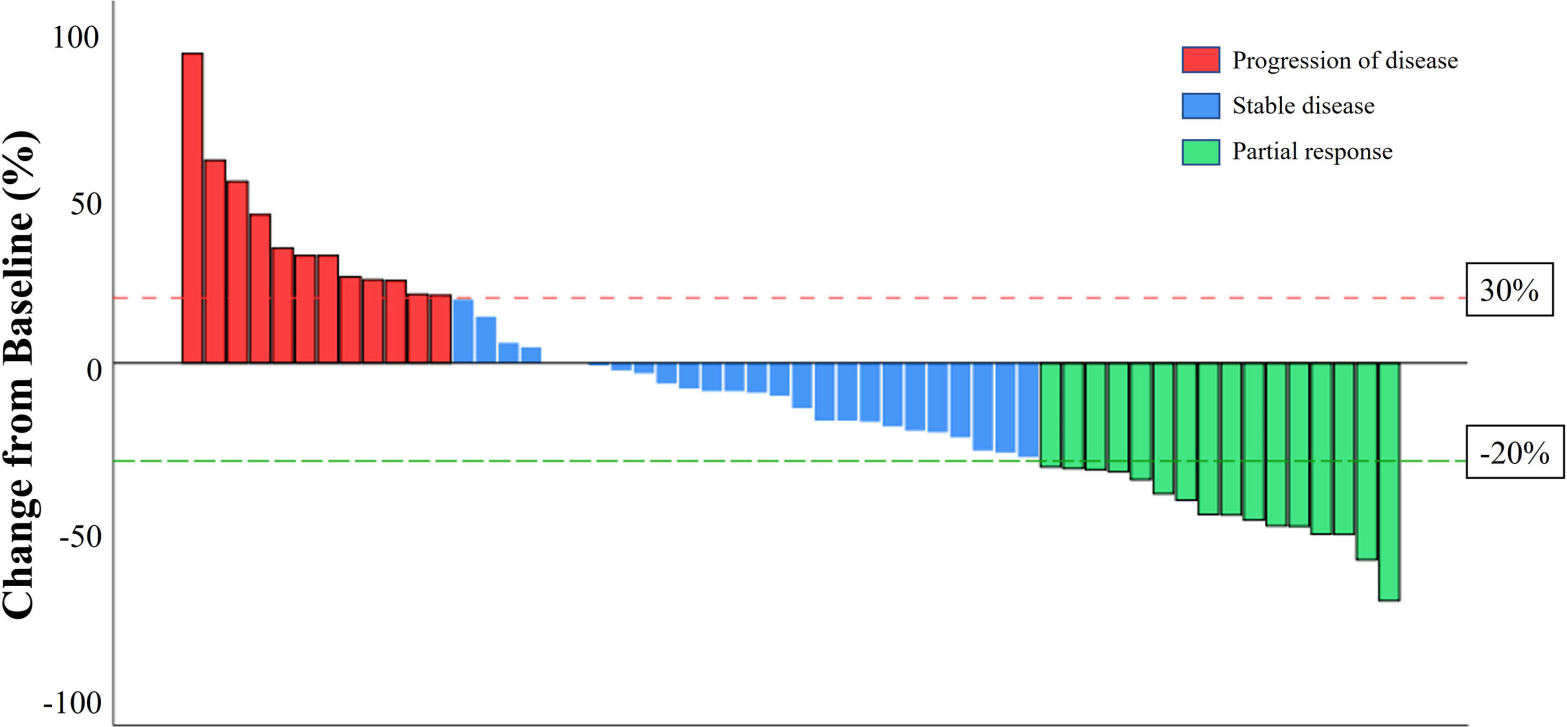
A waterfall plot of the best percentage change in the sum of the diameters of target lesions at any time in patient with measureable disease at baseline who received anlotinib-based treatment.

The median PFS of patients treated with anlotinib-based treatment was 5.7 months (95% CI, 3.17-8.23 months). In the subgroup, the median PFS was 11.7 months, 8.7 months, and 4.7 months in second-line, third-line and later-line treatment, respectively. The median PFS was 5.6 months, 5.7months, and 6.4 months in HER2+, HR+/HER2-, and TNBC patients, respectively (**Figure 1**). Of the total of 56 patients, no patients achieved CR, 16 (28.6%) had PR, 24 (42.9%) had SD, and 12 (21.4%) had PD. The ORR was 28.6% and the DCR was 71.4% (**Figure 2**). In terms of treatment regimes, most patients (91.1%) used anlotinib in combination with chemotherapy. The combination with capecitabine presented longer PFS (9.6 months, 95% CI, 8.35-10.85 months). Five TNBC patient used anlotinib plus immunotherapy, the median PFS was 10.2 months (95% CI, 0.53-19.87 months). The results are presented in **Table 2**.

**Table 2:** Efficacy of anlotinib-based therapy in 56 metastatic breast cancer patients.

Characteristics	Patients (n=56)	mPFS (month)	95% CI	P-value
**Molecular subtyping**				0.535
HER2+	14 (25.0%)	5.6	3.70-7.51	–
HER2-	42 (75.0%)	6.4	2.43-10.37	
HR+/HER2-	15 (26.8%)	5.7	1.81-9.59	–
Triple negative breast cancer	27 (48.2%)	6.4	1.03-11.77	–
**Treatment Line**				0.356
2	11 (19.6%)	11.7	0.00-26.88	–
3	15 (26.8%)	8.7	1.20-16.20	–
≥4	30 (53.6%)	4.7	2.41-8.23	–
**Disease type**				0.545
Visceral	43 (76.8%)	6.4	4.09-8.71	–
Non-visceral	13 (23.2%)	4.3	3.29-5.31	–
**Metastatic sites**				
Liver	22 (39.3%)	6.5	5.13-7.87	0.325
Lung	25 (44.6%)	6.5	1.31-11.69	0.924
Brain	15 (26.8%)	4.7	0.00-13.12	0.895
Bone	25 (44.6%)	4.0	2.46-5.54	0.058
Pleura	12 (21.4%)	3.6	1.39-5.81	0.046
**Prior therapies**				
Prior anthracyclines	49 (87.5%)	6.4	4.03-8.77	0.901
Prior taxane	56 (100%)	5.7	3.17-8.23	–
**Treatment regimens**				0.289
Combined with chemotherapy	51 (91.1%)	5.7	3.37-8.03	–
Capecitabine	16 (28.6%)	9.6	8.35-10.85	0.159
Taxane	15 (26.8%)	5.6	2.29-8.92	0.477
Vinorelbine	9 (16.1%)	3.6	1.34-5.86	0.004
Combined with immunotherapy	5 (8.9%)	10.2	0.53-19.87	–
**Starting dose**				0.464
12 mg	34 (60.7%)	6.5	0.70-12.30	–
10 mg	18 (32.1%)	4.3	3.16-5.44	–
8 mg	4 (7.1%)	6.4	–	–

mPFS, median progression free survival; CI, confidence interval.

We also analyzed the efficacy of patients with brain metastases. In our study, 15 patients had breast cancer with brain metastases diagnosed at baseline and 2 patients developed brain metastases during the treatment with anlotinib. Of the 15 patients with baseline brain metastases, nine patients had prior brain radiation therapy and six patients had brain radiation therapy within the past 6 months. A total of 14 patients had measurable brain lesions, 6 patients had SD, 5 patients had PR, and 3 patients had PD. One patient with non-measurable disease at baseline had SD. The ORR in central nervous system (CNS) was 33.3% and the DCR in CNS was 80.0%. The CNS PFS was 9.4 months (95% CI, 4.04-14.76 months). As for the six patients had brain radiation therapy within the past 6 months, the CNS ORR was 33.3%, and the CNS DCR was 66.7%.

### Adverse events

No patients experienced drug-related deaths. Five people (8.9%) discontinued the drug due to adverse reactions. Among them, grade 3-4 bone marrow suppression represented by neutropenia and thrombocytopenia are often the main reasons leading to dose reduction or drug withdrawal. Thrombocytopenia is the most common adverse event. Twenty patients (35.7%) had different degrees of platelet count decrease during the treatment of anlotinib. Among them, four patients (7.1%) had a grade 4 platelet count decrease. The platelet counts usually returned to normal after symptomatic treatment, including recombinant human thrombopoietin (rhTPO) and eltrombopag. Eleven people (19.6%) developed the hand-foot syndrome, and two (3.6%) of them developed grade 3 hand-foot syndrome. Gastrointestinal reactions such as anorexia (16.1%) and diarrhea (19.6%) are also common adverse reactions, but most patients deem these events tolerable. Other common adverse reactions include fatigue (17.9%), hypertension (16.1%), epistaxis (8.9%), and oral mucositis (7.1%) (**Table 3**).

**Table 3:** Adverse events that occurred in 56 patients with metastatic breast cancer during treatment with anlotinib-based therapy.

Treatment-related adverse events	All grades (%)	Grade 1-2 (%)	Grade 3-4 (%)
Platelet count decreased	20 (35.7%)	14 (25.0%)	6 (10.7%)
Hand-foot syndrome	11 (19.6%)	9 (16.1%)	2 (3.6%)
Diarrhea	11 (19.6%)	7 (12.5%)	4 (7.1%)
Fatigue	10 (17.9%)	10 (17.9%)	0 (0.0%)
Hypertension	9 (16.1%)	8 (14.3%)	1 (1.8%)
Anorexia	9 (16.1%)	9 (16.1%)	0 (0.0%)
Epistaxis	5 (8.9%)	5 (8.9%)	0 (0.0%)
Oral mucositis	4 (7.1%)	1 (1.8%)	3 (5.4%)
Neutrophil count decreased	5 (8.9%)	4 (7.1%)	1 (1.8%)
Lung infection	2 (3.6%)	0 (0.0%)	2 (3.6%)
Hemoptysis	2 (3.6%)	2 (3.6%)	0 (0.0%)
Hoarseness	2 (3.6%)	2 (3.6%)	0 (0.0%)
Blood bilirubin increased	2 (3.6%)	2 (3.6%)	0 (0.0%)
Anemia	1 (1.8%)	1 (1.8%)	0 (0.0%)
Erythra	1 (1.8%)	1 (1.8%)	0 (0.0%)

## Discussion

There is currently no standard treatment for patients with MBC who have progressed after standard treatment. The inhibition of tumor angiogenesis can effectively inhibit breast cancer growth in preclinical studies (3). Monoclonal antibodies represented by bevacizumab have shown certain effects in clinical application. E2100, AVADO and RIBBON-1 trails confirmed that bevacizumab combined with chemotherapy can improve patients’ PFS and ORR, but not overall survival (OS) (16–18). However, in the RIBBON-2 study, the use of bevacizumab combined with chemotherapy in second-line and beyond patients showed no benefit in PFS, ORR or OS (19). Taking the inconsistent efficacy and relatively high rates of adverse events into account, U.S. Food and Drug Administration (FDA) revoked the approval of bevacizumab with paclitaxel for the treatment of women with HER2-negative MBC. In the studies of anti-angiogenic TKIs, the efficacy also remains controversial. The SOLTI-0701 study reported PFS benefit for sorafenib plus capecitabine as first- or second-line treatment, and the NU07B1 study showed a not statistically significant increase in PFS when combined with first-line paclitaxel (20, 21). With all that in mind, the efficacy is still ambiguous in clinic. Anlotinib is a new type of anti-angiogenic TKI. Yuan’s phase II study of anlotinib in pre-treated HER2 negative MBC showed that in patients had treatment failure after at least one prior chemotherapy regimen in the metastatic setting, the median PFS for anlotinib was 5.22 months (13), indicating a potential new treatment option for breast cancer. Thus, we began to try adding anlotinib to doctor’s choice of treatment, and we have indeed found that it has a good effect on some patients. However, few research on the efficacy of the clinical practice of anlotinib in MBC patients has been made. We hope that this retrospective study can provide clinicians with more clinical data about this drug and a new perspective for the treatment of MBC.

The median PFS time of a total of 56 patients was 5.7 months (95% CI, 3.17-8.23 months). In second-line, third-line, and beyond treatment, the median PFS was 11.7 months, 8.7 months, and 4.7 months, respectively. Since our study was a single-arm retrospective study with no control group, we investigated the previous single-arm retrospective study on MBC patients in our hospital. In the clinical study of platinum-based chemotherapy (not including anlotinib) for advanced MBC, the median time to progression (TTP) of second and third-line treatment was 6.0 and 3.7 months (22).

In our study, the median PFS of anlotinib-based treatment in HR+/HER2- and HER2+ patients are 5.7 months and 5.6 months while the median PFS of TNBC patients is 6.4 months. We believed that anlotinib may be more effective in TNBC. However, due to our small sample size, this hypothesis cannot be demonstrated through statistical data for the time being. The addition of angiogenic therapy extends PFS and OS in TNBC subgroup analysis of RIBBON-2 trial (23). We hope that prospective studies with large samples can help us further improve. TNBC lacks therapeutic targets. Although immunotherapy and PAPR inhibitors have changed the treatment pattern of metastatic TNBC in recent years, the efficacy of immunotherapy remains controversial and PAPR inhibitors are only suitable for patients with BRCA mutations. Currently, sacituzumab govitecan can be considered in metastatic TNBC with a median PFS of 5.6 months in patients have received at least one prior treatment for advanced disease (24). According to our study, anlotinib-based therapy showed comparable efficacy in TNBC with the median PFS of 6.4 months in second-line or beyond treatment. Although there is no randomized controlled trial comparing anlotinib versus sacituzumab govitecan, considering the high price and poor accessibility of sacituzumab govitecan in Chinese patients, anlotinib is more feasible for Chinese patients.

In terms of treatment regimes, 51 patients were treated in combination with chemotherapy. Among these patients, the capecitabine team showed better PFS benefit. The median PFS of capecitabine and anlotinib reached 9.6 months. Capecitabine reversed the inhibitory effects of tumor angiogenesis and tumor growth under anti-VEGF antibody treatment (25). The satisfying results of capecitabine and anlotinib may be attributed to the synergistic anti-angiogenesis effect of these two drugs. In addition, both anlotinib and capecitabine are oral drugs and the patients’ compliance will be improved. We also found that the median PFS of five TNBC patients who were treated with anlotinib combined with immunotherapy reached 10.2 months. Studies have shown that anlotinib can inhibit tumor growth by down-regulating the expression of programmed cell death ligand 1(PD-L1) on vascular endothelial cells, thereby improving the immune microenvironment (26). Synergistic antitumor therapeutic effect has been seen when anlotinib is used in combination with PD-1/PD-L1 blockade (27). The FUTURE-C-PLUS study also showed encouraging results of camrelizumab in combination with famitinib and nab-paclitaxel in TNBC (28). The regimen of anlotinib combined with immunotherapy deserves further explorations in TNBC patients based on our current findings.

Due to the existence of the blood-brain barrier, water-soluble and large anticancer agents have limited efficacy in the treatment of brain metastases. Angiogenesis inhibitors have showed some effect in the treatment of brain metastases (29). In NSCLC, anlotinib already showed improvement in the intracranial local control in patients with brain metastases (30). In the Phase III NALA Trial, the CNS ORR was 32.8% for neratinib plus capecitabine and 26.7% for lapatinib plus capecitabine (31). The intracranial PFS was about 4.2 months in HER2 positive MBC, and the addition of tucatinib improved intracranial PFS to 9.9 month in the HER2CLIMB study (32). In our study, for patients with brain metastases, the CNS ORR was 33.3%, the CNS DCR was 80.0%, and the median CNS PFS was 9.4 months. The addition of antiangiogenic drugs to the treatment of patients with brain metastases may be an effective means of controlling brain lesions. We still need to mention that 6 patients (40%) had received partial or whole brain radiation within 6 months before receiving anlotinib, which had an impact on our results.

Bone marrow suppression, represented by thrombocytopenia, is the most common adverse event and the leading cause of drug dose adjustment. It can often be controlled at an early stage through timely treatment due to periodic inspection of blood routine examination. Gastrointestinal reactions are also common AEs. Some patients experience a certain loss of appetite and/or mild to moderate diarrhea. Some patients complain of nosebleeds during the early stages of anlotinib treatment. The previous meta-analysis presented an increase in the incidence of bleeding events in patients with VEGFR TKIs compared to placebo treatment (33). In our study, bleeding symptoms are relatively light and can often be relieved after simple procedures such as compression. Hand-foot syndrome is usually found in patients with the treatment in combined with capecitabine. In summary, with regular blood routine examination, anlotinib is relatively safe, and most adverse events are manageable.

The main limitation of our study is that this is a single-center retrospective study with a small sample size. Due to the short follow-up time, we cannot prove whether anlotinib can affect the overall survival rate of MBC patients.

## Conclusion

Anlotinib showed good efficiency and manageable toxicity in metastatic breast cancer patients with failure of standard treatment. It is emerging as a treatment option for metastatic breast cancer, but its efficacy needs to be further proven through prospective studies involving more patients.

## Data availability statement

The original contributions presented in the study are included in the article/supplementary material. Further inquiries can be directed to the corresponding authors.

## Ethics statement

The studies involving human participants were reviewed and approved by the ethics board of Jiangsu Cancer Hospital. Written informed consent for participation was not required for this study in accordance with the national legislation and the institutional requirements.

## Author contributions

Conception and design: YY; Administrative support: LZ; Provision of study materials or patients: LZ; Collection and assembly of data: YQ and HZ; Data analysis and interpretation: YQ and KL; Manuscript writing: All authors; Final approval of manuscript: All authors.
